# The association of perceived leadership style and subjective well-being of employees in a tertiary hospital in Germany

**DOI:** 10.1371/journal.pone.0278597

**Published:** 2022-12-13

**Authors:** Rebecca Erschens, Tanja Seifried-Dübon, Felicitas Stuber, Monika A. Rieger, Stephan Zipfel, Christoph Nikendei, Melanie Genrich, Peter Angerer, Imad Maatouk, Harald Gündel, Eva Rothermund, Martin Peters, Florian Junne

**Affiliations:** 1 Department of Psychosomatic Medicine and Psychotherapy, University Hospital Tuebingen, University of Tuebingen, Tuebingen, Baden-Wuerttemberg, Germany; 2 Institute of Occupational and Social Medicine and Health Services Research, University Hospital Tübingen, Tuebingen, Baden-Wuerttemberg, Germany; 3 Department for General Internal Medicine and Psychosomatics, University Hospital Heidelberg, Heidelberg, Baden-Wuerttemberg, Germany; 4 Institute of Psychology, Work and Organisational Psychology, University of Duisburg-Essen, Essen, Nordrhein-Westfalen, Germany; 5 Institute of Occupational, Social and Environmental Medicine, Centre for Health and Society, Faculty of Medicine, Heinrich-Heine-University Düsseldorf, Düsseldorf, Nordrhein-Westfalen, Germany; 6 Section of Psychosomatic Medicine, Psychotherapy and Psychooncology, Department of Internal Medicine II, Julius-Maximilian University Würzburg, Würzburg, Bayern, Germany; 7 Department of Psychosomatic Medicine and Psychotherapy, University Hospital Ulm, Ulm, Baden-Wuerttemberg, Germany; 8 Department of Psychiatry II, Ulm University, Günzburg, Bayern, Germany; 9 Department of Psychosomatic Medicine and Psychotherapy, Otto von Guericke University Magdeburg, University Hospital Magdeburg, Magdeburg, Sachsen-Anhalt, Germany; Osaka University, JAPAN

## Abstract

**Background:**

Professionals in the healthcare sector are a particularly vulnerable group for occupational strain due to high work-related psychological stress. For the implementation of targeted stress-prevention interventions as an important part of a workplace health management programme for all occupational groups and hierarchy levels, information about the current state of their mental health is mandatory. Hence, this study investigated the association of general well-being and different leadership styles among employees in a German tertiary hospital.

**Methods:**

Via an online survey, 10,101 employees were contacted. The final sample consisted of 1137 employees. Of these, 27.7% described themselves as leaders and 72.3% as followers. Most participants were female (74.8%), more than half were under 41 years old. Besides control variables, general well-being (WHO-5) and leadership style (transactional and transformational, laissez-faire and destructive leadership) were assessed.

**Results:**

Leaders reported higher well-being scores than followers. Physicians without leadership responsibilities had the lowest scores for well-being. Practitioners of both transformational and transactional leadership were associated with higher well-being scores, while those practicing laissez-faire and destructive leadership had lower scores for almost every professional group.

**Conclusion:**

Results highlight the necessity for future multimodal health-preventive leadership interventions feature behavioural and organizational intervention modules specifically tailored to hospital professionals at different hierarchical and functional levels to foster the mental health of employees.

## Introduction

At a tertiary hospital, a variety of occupational groups gather under one roof: first and foremost are the physicians and nursing staff, but there are also administrative staff, IT staff, clinical services, office assistants, scientists, and therapeutic professionals. All these groups not only share the same senior management and conjointly pursue the goal of excellent patient care, but they also are all subject to the work stress that arises through a highly complex workflow and constantly intensifying work processes at the hospital. Work stress functions as a risk factor for the development of mental and (psycho)somatic disorders like, for example, burnout (e.g. [1]), depression (for an overview see [2]), anxiety disorders (e.g. [3]), musculoskeletal disorders [4] or cardiovascular disorders (e.g. [5]). In this context, it is not surprising that in the German health sector the proportion of work incapacity cases due to mental diagnoses is almost three times higher (16.1 incapacity cases per 100 people) than, for example, in the agricultural and forestry sector (5.8 incapacity cases per 100 people [6] (p. 368)). Note that the most concerned sectors following healthcare are public administration and social security (second) and education (third).

Promoting and maintaining the health of employees at the hospital workplace is important for organisations, considering legal requirements and individual well-being, as well as increasing employee motivation and performance, and employer attractiveness [7]. Workplace-health management in Germany is a holistic strategy designed to protect, promote and manage the health of employees in the workplace. It consists of four key aspects: (1) occupational health and safety measures, (2) reintegration management, (3) workplace health promotion (WHP), and (4) appropriate human resource development [8]. The maintenance and fostering of employees’ health presents itself as an economic necessity (only healthy employees can perform their work adequately and the shortage of health workers may give hospitals which focus on their employees’ health a competitive advantage) and a humanitarian imperative (as we already outlined in Seifried-Dübon et al. [9]).

The study presented here is part of the multicentre project “Mental health in the workplace hospital” (*Seelische Gesundheit am Arbeitsplatz Krankenhaus*; acronym: SEEGEN) funded by the German Federal Ministry of Education and Research (BMBF, FKZ 01GL1752C). In SEEGEN, the primary objective is to improve mental health in the hospital workplace with a complex intervention within a Randomized Control Trial (RCT) consisting of five interventions to promote different thematic priorities for hospital employees: (i) Top Management Training, (ii) Promoting Stress-Preventive Relational Leadership Competence, (iii) Dilemma Competency–Coping by Taking Responsibility, (iv) Reconciling Work and Family Life and (v) Staying Healthy at Work. The intervention on stress-preventive leadership is mainly based on the concept of transformational leadership (see [10]). SEEGEN aims to improve the mental health of an entire organisation and can be understood as a complex intervention covering the third key aspect of workplace health management, WHP, as health-related topics are addressed together with the improvement of working conditions (the first key aspect of occupational health and safety measures) because social relations and structural prevention at the workplace should be improved as well as human resource development (the fourth key aspect).

The presented study is an anonymous general survey at a representative and large tertiary hospital in Germany which is embedded within the SEEGEN study as an additional subproject. The purpose was designed to obtain perceptions on the topics of leadership behavior, relationship quality, stress level and well-being of followers and leaders. Simultaneously, the relevant evidence gained here was directly incorporated into the further design of the SEEGEN RCT and its interventions.

Mental health has been conceptualized as a continuum (for an alternative two continua model, see for example [11]) which extends from negative to positive mental health states (see [12], based on WHO’s definition [13]). Negative states can be described as the absence of symptoms like burnout, depression or other health complaints, while positive states are described as psychological functioning or well-being. In more detail, WHO defines mental health as “a state of well-being in which every individual realizes his or her own potential, can cope with the normal stresses of life, can work productively and fruitfully, and is able to make a contribution to her or his community” [14]. This definition also emphasises that mentally healthy employees will work more productively (happy worker–productive worker thesis [15]; for a review see [16]), which is in the interest of the direct leader as well as the whole organization. In their recent work, Capone and colleagues [17] reinforce the approach of considering the concept of well-being as a positive individual outcome for employees’ life (e.g. general mental health) on the one hand, and job satisfaction as an important domain specific organizational outcome on the other. The WHO also distributes a questionnaire for assessing subjective psychological well-being: the WHO-5 well-being index, a widely used scale of only five items with adequate validity as a screening tool [18]. Since it is important for the implementation of stress-preventive measures in terms of WHP and specific for the SEEGEN RCT to know the current health status, we used the WHO-5 in this study to determine the well-being of employees (leaders as well as followers) at a tertiary hospital in Germany.

**Research Question 1:** What is the state of well-being for leaders and followers at a tertiary hospital in Germany? How does it differ between the three largest occupational groups (i.e. physicians, nursing staff, administrative staff)?

To enable the development, implementation, and evaluation of relevant prevention programmes fostering well-being, theoretical knowledge about how work stress comes about–in the health sector and elsewhere–is indispensable. The three most common theoretical models which offer approaches to the development of work stress are the job demand–control model [19], the organizational Justice model [20] and the model of effort–reward imbalance (ERI) [21]. Junne et al. [22] has suggested the social dimension as a cross-sectional area to these models. Specifically, social relations at the workplace and leadership quality are the stress dimensions in work settings that health-care professionals who regularly encounter mentally strained employees–primary care physicians, occupational health physicians, psychotherapists and human resource managers–rate highest for relevance to the development of psychological distress and associated mental disorders [23]. Corroborating this claim, a recent prospective study by Schmidt et al. [24] showed that a lack of supportive leadership can predict suboptimal self-rated health even after 10 years of follow-up, even after “controlling for self-rated health, job strain and other risk factors at baseline” [24] (p. 6). Further support is given by Schyns and Schilling [25] (p. 138) when they sum up the research result for so-called “bad leaders” (cf.” How bad are the effects of bad leaders? A meta-analysis of destructive leadership and its outcomes”) by presenting meta-analytic data reporting positive correlations of destructive leadership with, for example, resistance towards the leader and turnover intention, and negative correlations with, for example, individual performance and well-being (for behavioural examples of destructive leadership and laissez-faire leadership, see Table 1).

**Table 1:** Behavioural examples for transformational (TF) and transactional (TA) subscales.

Subscale	Behavioural examples
Innovation	The leader challenges employees to rethink their assumptions about their work and how to cope with them; helps employees to be creative and innovative.
Team spirit	The leader supports cooperation and team spirit among employees; she encourages and emphasizes that employees should work for a common goal.
Performance	The leader has high expectations of the quality of work and expects high performance from the employees while also communicating her trust in the capability of employees to reach this performance.
Individuality	The leader shows respect for her employees, holds them in high esteem, takes their individual needs into account and distributes tasks according to the developmental needs of the employees.
Vision	The leader generates new opportunities for the team; she develops clear and colourful visions of the future and the meaning of work and also shows how these can be realized.
Role model	The leader acts as a role model for the employees; she shows the behaviour that she also expects from her employees.
Objective	Leader and employees negotiate goals together and it is clarified what the employees can expect when goals are achieved (e.g. praise, feedback).
Management-By-exception	The leader only intervenes when employees’ performance declines or problems occur, i.e. she does not deal with the problems proactively but reacts when certain processes no longer work.
Laissez-faire (LF)	The leader does not intervene in the work processes of employees but rather leaves them to themselves.
Destructive (D)	The leader displays destructive behaviour such as hostility, non-compliance with agreements or the impulsive release of negative feelings.

Thus, on the one hand, leadership can act as a stress factor for employees, while, on the other hand, support from the leader can act as a resource and protective factor for mental health. Concerning mental distress (measured through symptoms of anxiety and depression), Finne et al. [26] found support from the immediate superior, fair leadership, and positive challenge to be the most consistent protective factors in their follow-up study at an interval of two years after a baseline survey. To sum up, when thinking about the organizational prevention of work stress and the fostering of mental health, the role of direct leaders and supervisors is an important one. Extensive research results support the positive association of various stress-preventive leadership behaviours with well-being (e.g. [27]; for reviews and meta-analyses, see [12, 28–30]).

One leadership approach that has been repeatedly shown to foster employees’ well-being is the transformational leadership approach (e.g. [31, 32]). Transformational leadership and its conceptual partner transactional leadership were first introduced by Burns [32] (original work from 1978) and further elaborated by Bass and colleagues (e.g. [33, 34]; for overviews see [31, 35]). Transactional leadership gets its name from the ‘transactions’ between leader and employees–time and performance, for example, are exchanged for salary, appreciation, bonuses etc. The leader and her followers are focused on their own individual goals and not much more. The two subscales of transactional leadership are contingent reward and management by exception (for examples of the respective leadership behaviour, see Table 1). *Transformational leadership* is described as a leadership style through which the individual values and goals of employees might get “transformed” over time so that they eventually match the goals of the team or organization. While the original conceptualization by Bass and colleagues differentiated between four key concepts, the research group around Podsakoff [36, 37] introduced a conceptualization of six key concepts which were recently incorporated into a new German questionnaire by Rowold and Poethke [38] called *Fragebogen zur Integrativen Führung* (FIF) (Questionnaire on Integrative Leadership). These six concepts are fostering innovation, team spirit development, performance development, individuality focus, providing a vision and being a role model (see Table 1). Transformational leadership has been shown to be associated with positive outcomes on the part of the employees such as work motivation (e.g. [39]), organizational citizenship behaviour (i.e. performing outside of the actual job description, e.g. [40]), job satisfaction (e.g. [41]) and, of course, well-being (e.g. [12, 28]), even though the association is often mediated by other variables from the work context (e.g. [42]). Similar positive effects have been reported for the contingent reward scale (note that the objective scale of the FIF is comparable content-wise to what other instruments measure as contingent reward) as part of transactional leadership (e.g. [39, 43]), while the results for management-by-exception are mixed and sometimes even the opposite (e.g. [39, 44]). Our second research focus was therefore on the association between leadership styles and followers well-being at a German tertiary hospital.

**Research Question 2:** How are perceived leadership styles and subjective well-being at this tertiary hospital associated with each other?

## Materials and methods

Please note that parts of this study have already been published elsewhere and that therefore this method section resembles in part the method section in Stuber et al. [45].

The study was approved by the ethics committee of the University Hospital and Medical Faculty of the University of Tübingen (622/2017BO2) and was carried out in accordance with the recommendations of the ICH-GCP guidelines, Declaration of Helsinki in its current version (Fortaleza 2013). All subjects gave written informed consent in accordance with the Declaration of Helsinki (Fortaleza 2013).

### Participants and procedure

The study participants were employees at a tertiary hospital in Germany who were invited to complete a cross-sectional online survey via email. Having been approved beforehand by the chief executive board and the employees’ council of the participating tertiary hospital, the survey was active from the 23rd of May until the 18th of July 2018. The invitation to the online survey was sent by the executive board to an ‘all-users’ email list of the tertiary hospital which consisted of 10,101 individual email addresses. The subsequent response rate was 11.26%.

Email addresses wishing to respond clicked on a link in the invitation email which would redirect them to a web page of the Unipark programme of the EFS Survey Software [46] which we used for questionnaire handling. Participants were greeted by the study information which informed them that their participation in the study would take 8 to 12 minutes (which would count as working time) and was voluntary and anonymous and was backed by the employees’ council. They were also informed that results of this study could be presented to the executive board and the employees’ council as well as being used for scientific publications. Further relevant information on data security and contact persons was given. Employees received no reward for their participation. Upon their consent to participate in the study, the actual questionnaire was administered, which is described in the Measures section. The final sample consisted of 1137 employees, of which 27.7% self-identified as leaders and 72.3% as followers without leadership responsibilities. Of all the employees, 62.1% worked full-time. Most of the followers were female (74.8%) and a little more than half of the followers were younger than 41 years (51.2%). Amongst the leaders, 59.6% were female and 59.6% were older than 45 years. The occupational groups with the largest participation rates were administration (19.6%), nursing staff (18.5%) and physicians (13.7%). For more details on the sample, see Stuber et al. [45].

### Measures

#### Leadership

We applied Module A and D of the standardized “Questionnaire on Integrative Leadership” (Fragebogen zur Integrativen Führung (FIF [38])) in order to assess transactional and transformational leadership (Module A) as well as laissez-faire and destructive leadership (Module D, negative leadership). Followers assessed their direct leader on a five-point Likert scale (ranging from 1 = “fully disagree” to 5 = “fully agree”). Eight items assessed transactional leadership (e.g. “My leader clarifies what I can expect as reward or appreciation for successful work performance”), consisting of the two scales contingent reward and management by exception. Transformational leadership was assessed by 24 items (e.g. “My leader knows my individual interests and personal goals”), consisting of the six scales–innovation, team spirit, performance development, focus on individuality, vision and role modelling. Laissez-faire was measured by four items (e.g. “My leader postpones the answering of important questions”), as was destructive leadership (e.g. “My leader takes his or her negative feelings (rage, anger, frustration) out on me”). Please note that in the original German version the employed term for leader, that is, „die Führungskraft“, is feminine so that the possessive pronoun would always read “her.” Internal consistency, that is, Cronbach’s α of the relevant scales varied in the validity studies of this questionnaire between .79 (management by exception) and .92 (team spirit and role modelling).

#### Well-being

We assessed well-being with the five-item World Health Organization well-being index (WHO-5 [47, 48]) in its German version [49]. The WHO-5 (for an overview see [18]) is a standardized unidimensional scale of five positively formulated items (e.g. “Over the last two weeks I have felt cheerful and in good spirits”) that have to be rated by participants on a six-point Likert scale (ranging from 5 = “all of the time” to 0 = “at no time”; a percentage score is obtained by multiplying by 4 so that the overall score ranges from 0 to 100). It measures subjective psychological well-being over the last two weeks and the cut-Off score of 50 can be used for screening for clinical depression [18]. Internal consistency in the German validation study by Brähler et al. [49] amounted to Cronbach’s α = .92. The Cronbach’s α of the WHO-5 and the FIF of this study are reported below in the Results section.

### Statistical analyses

Missing data were scarce, and we decided not to compute scale values when an item was missing. In the statistical analyses, missing data were deleted pairwise. With respect to transparency, we report the included number of participants (*n*) for each analysis. Further calculations are based on the assumption of approximative normal distribution. For comparing the well-being of leaders and followers overall, as well as for the three largest occupational groups, we used a t-test and an ANOVA. To analyse differences concerning the cut-off score of the WHO-5, *χ^2^* tests were applied. To examine Research Question 2, we calculated correlations and conducted a multiple linear regression analysis. All statistical analyses were conducted with IBM SPSS version 25 [50].

## Results

Before the association between leadership style and well-being is reported, this section focuses on the state of well-being for the staff at the tertiary hospital first.

### Well-being

**Fig 1:**
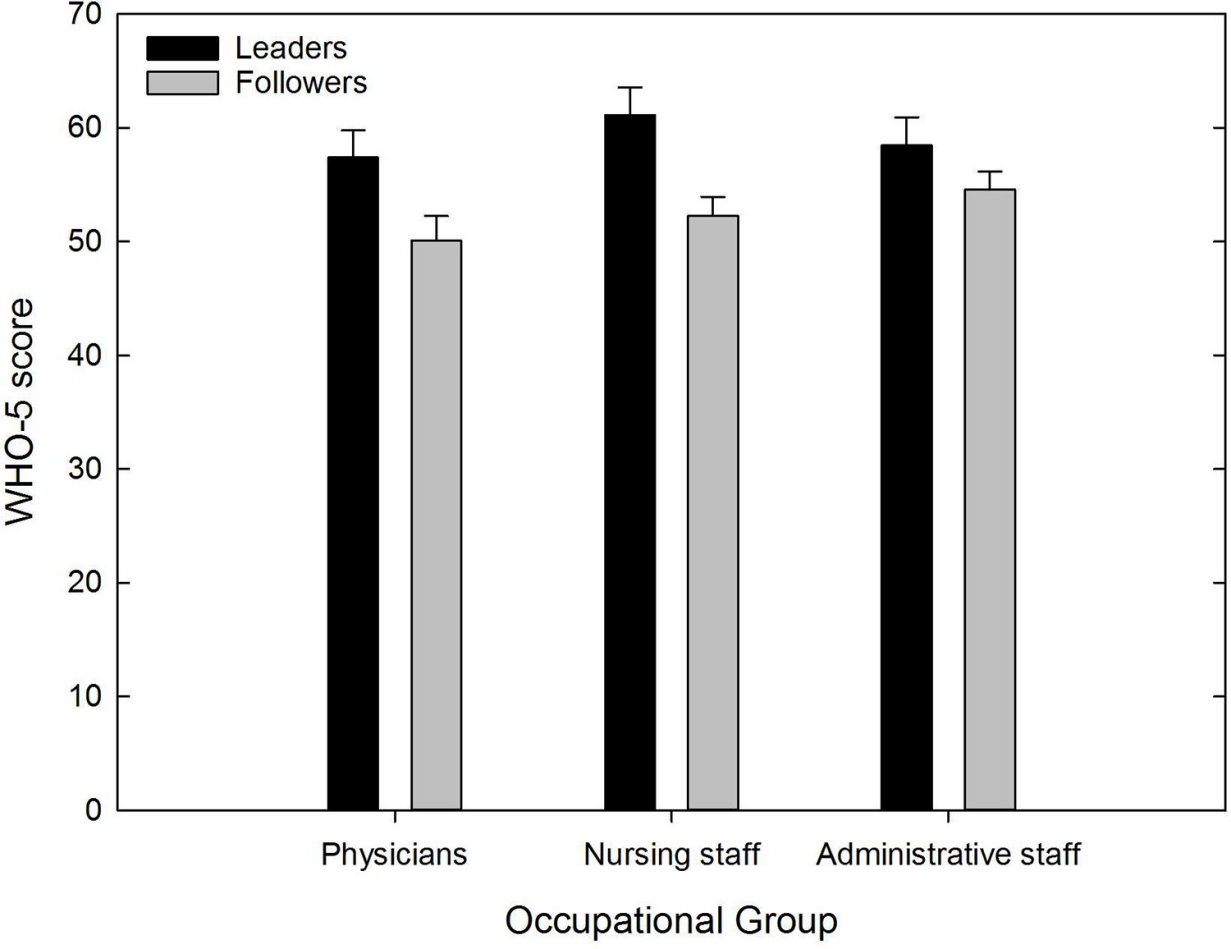
WHO-5 scores for occupational group and hierarchy level. Error bars represent the standard error (min = 0, max = 100).

First, we submitted the WHO-5 percentage scores of leaders and followers to an independent t-test, which revealed the mean WHO-5 score of leaders (*M* = 56.8, *SD* = 19.2, *SEM* = 1.1) was larger than that of followers (*M* = 52.1, *SD* = 21.3, *SEM* = 0.8), *t*(606.1) = -3.47, *p* = .001 (two-tailed), Cohen’s d = 0.23. Second, we categorised leaders and followers according to whether their WHO-5 score was > 50 or ≤ 50. Subsequently, a *χ^2^*-test on cutoff and hierarchical position (leader vs. followers) test revealed that followers were more likely to have a WHO-5 score ≤ 50 than leaders, *χ^2^*(1) = 9.65, *p* = .002, *φ* = -.09. The odds ratio–that is, the risk that stems from being a follower–to have a WHO-5 score ≤ 50 is 1.54. To take a closer look at well-being differences in the three largest occupational groups, that is, physicians, administration, and nursing staff (please note that all other occupational groups were left out of the following analysis, so that the *n* of this subsample is 575), we first submitted the WHO-5 scores to a two-way ANOVA with the two between-subject factors being hierarchy level (leaders vs. followers) and occupational group (physicians vs. administration vs. nursing staff). The main effect of hierarchy level reached significance, *F*(1, 569) = 14.33, *p* < .001, but neither the occupational group nor the interaction (Fig 1) did. Second, we submitted the data of each group to a separate *χ^2^*-test regarding cutoff and hierarchical position. For the physicians, the followers were not more likely to have a WHO-5 score ≤ 50 than the leaders, *χ^2^*(1) = 3.43, *p* = .06, *φ* = -.15, although statistical significance was just missed by a narrow margin; the odds ratio was 1.88. For the nursing staff, followers were more likely to have a WHO-5 score ≤ 50 than leaders, *χ^2^(*1) = 8.91, *p* = .003, *φ* = -.21; the odds ratio was 2.59. For the administrative staff, neither followers nor leaders were more likely to have a WHO-5 score ≤ 50, *χ^2^*(1) = 2.11, *p* = .147, *φ* = -.10; the odds ratio was 1.57.

### Leadership styles and well-being

Please note that the following analyses only incorporate the **data of the followers** since we analysed the association between the subjective well-being of followers and their subjective perception of their leader’s behaviour.

### Report of correaltions between well-being subjective perception of leader’s behaviour

Table 2 reports the descriptive values (i.e. *M*, *SD*, and *n*) alongside the intercorrelations of leadership scales, the correlations of leadership scales with well-being and Cronbach’s α for each leadership scale. As can be seen, all leadership scales as well as the WHO-5 scale reached at least a sufficient Cronbach’s α between 0.83 and 0.97. Transformational leadership (TF) and transactional leadership (TA) are positively correlated with WHO-5 with *r* = .36 (TF) and *r* = .25 (TA), while laissez-faire (LF) and destructive leadership (D) are negatively correlated with WHO-5 with *r* = -.24 (LF) and *r* = -.26 (D).

**Table 2:** Descriptive statistics and correlations between WHO 5 and the leadership scales.

	Descriptive Statistics			Correlations for each Leadership Scale				
Scale	M	SD	N	TF	TA	LF	D	WHO-5
Transformational leadership (TF)	2.86	1.04	737	**.97**				
Transactional leadership (TA)	2.69	0.84	737	.70**	**.83**			
Laissez-faire (LF)	2.38	1.18	804	-.64**	**-.**44**	**.89**		
Destructive leadership (D)	1.88	1.04	780	-.61**	-.29**	.55**	**.87**	
Well-Being (WHO-5)	52.14	21.29	803	.36**	.25**	-.24**	-.26**	**.86**

*Note*. The diagonal numbers in **bold font** represent **Cronbach’s α. Correlations** with ** are significant with α = .001, two-tailed.

### Report of correlations between leadership styles and WHO-5 for each occupational group

For a more detailed look with respect to the occupational groups, Table 3 presents the correlations between leadership styles and WHO-5 separately for each occupational group (please note that clinical services were incorporated into the “others” category since this group contained only eight participants). With respect to the mean scores for the leadership styles, there are no significant differences between the occupational groups with the following order of rating: TF, TA, LF and D. Some clear differences between occupational groups and leadership styles can be observed:

- *scientists* showed the highest correlations for transformational leadership (r = .49) and laissez faire (r = -.38)
- Transactional leadership held the highest correlation for *therapeutic staff* (r = .42)
- Destructive leadership held the highest correlation for the *IT staff* with r = -.47
- For some occupational groups, correlations remained insignificant: e.g. transformational leadership for physicians and IT staff; transactional leadership for physicians, IT staff and office assistants; laissez faire for physicians, therapeutic staff and office assistants; and destructive leadership for physicians. No correlation was significant for physicians.

**Table 3:** Descriptive statistics and correlation with WHO-5 for each leadership scale and occupational group.

		Leadership Scale			
Descriptive Statistics	Occupational group	TF	TA	LF	D
M	Physicians	2.82	2.82	2.33	2.05
	Nursing staff	2.84	2.60	2.40	1.89
	Therapeutic staff	2.78	2.51	2.20	1.77
	Administrative staff	2.81	2.72	2.46	1.89
	IT	3.06	2.72	2.41	1.73
	Office assistants	2.82	2.64	2.28	1.87
	Scientists	3.16	2.92	2.43	1.85
	Others	2.74	2.58	2.40	1.91
SD	Physicians	0.95	0.79	1.21	1.14
	Nursing staff	1.08	0.91	1.26	1.10
	Therapeutic staff	0.91	0.75	1.05	0.91
	Administrative staff	1.07	0.87	1.21	1.07
	IT	0.93	0.84	0.98	0.91
	Office assistants	1.08	0.84	1.10	0.97
	Scientists	0.99	0.72	1.25	1.07
	Others	1.11	0.81	1.16	1.04
n_Δ_	Physicians	78	81	82	81
	Nursing staff	127	131	141	134
	Therapeutic staff	55	51	56	56
	Administrative staff	134	142	154	149
	IT	51	51	55	52
	Office assistants	91	95	97	94
	Scientists	79	85	85	85
	Others	122	121	134	129
R_†_	Physicians	.15	.03	-.01	-.03
	Nursing staff	.23**	.24**	-.22**	-.26**
	Therapeutic staff	.30*	.42**	-.07	-.28*
	Administrative staff	.44**	.32**	-.30**	-.28**
	IT	.24	.16	-.34*	-.47**
	Office assistants	.42**	.19	-.14	-.31**
	Scientists	.49**	.36**	-.38**	-.28*
	Others	.46**	.30**	-.32**	-.29**

*Note*. n_Δ_ denotes the sample size for calculating M and SD. Correlations with * are significant with α = .05, two-tailed; those with ** are significant with α = .001, two-tailed. † R represent the exact correlation with WHO-5 for each Leadership Scale and Occupational Group.

### Regression analysis

To investigate how much variance of well-being the different leadership styles can explain in this specific sample, we conducted a multiple regression analysis. We decided to omit the physicians, since their data did not show any correlations between leadership styles and well-being and would therefore bias the regression analysis.

In the first step, we entered the gender of the participant as well as the gender of the assessed leader as control variables into the regression analysis. Gender differences in well-being have been described in research, even though results are rather mixed (see for example [51]), and, as Batz and Tay [51] (p.11) state “it depends”. Furthermore, Eagly et al. [52], for example, have shown that female leaders lead more transformationally than male ones, which is why the gender of the leader was included as a control variable. In addition to these two variables, occupational group was entered as a control variable. Since occupational group was manifold, we needed to recode it as a dummy variable: Administrative staff was chosen as the baseline group (it was the largest group), and each dummy variable corresponding to the other occupational groups was defined as the difference in well-being for the administrative staff and every other occupational group (for an overview on dummy variables see [53]; pp. 208–215). In a second step, transformational leadership, transactional leadership, laissez-faire leadership, and destructive leadership were all included simultaneously. The assumptions of multiple linear regression have generally been met: regarding independence of residuals, the Durbin–Watson statistic was 1.79; homoscedasticity as well as a normal distribution of residuals could be observed; values of residuals were normally distributed, as the P–P plot showed; there was no multicollinearity (all correlations < .8, all tolerance values > .29 and all VIF < 3.5); Cook`s distance values were all under 1; the relationships between predictors and the dependent variable were, however, only slightly linear.

Regression analysis showed that the overall variance of well-being that could be explained by the control variables was *R^2^* = 0.02, *F*(8, 589) = 1.84, *p* = .067 in Model 1. The variance that could be explained by Model 2, which included transformational, transactional, laissez-faire and destructive leadership was *R^2^* = 0.16, *F*(4,585) = 26.58, *p* < .001. This means that the additional inclusion of the leadership variables added 14% of variance explanation compared with Model 1 which included only the control variables.

The coefficients for the various predictors in Model 2 are depicted in Table 4. A significant contribution to variance explanation can be detected for the control variables Admin vs. Therapeutic and Admin vs. IT (*p* = .049 and *p* = .007) and for transformational leadership as far as the predictors are concerned (*p* < .001). Transformational leadership had by far the largest beta coefficient (.30).

**Table 4:** Coefficients of multiple linear regression analysis for followers’ well-being measured with WHO-5 Model 2 incorporating control variables as well as predictors.

Variables	B	SE(B)	β	T	P	CI(B)
Constant	35.75	6.70		5.34	< .001	22.59–48.92
Gender	1.09	2.09	.02	0.53	.600	-3.00–5.19
Gender leader	0.68	1.73	.02	0.39	.696	-2.72–4.07
Admin vs. Nursing	-2.14	2.61	-.04	-0.82	.411	-7.26–2.98
Admin vs. Therapeutic	6.68	3.38	.08	1.98	**.049** ^1^	0.04–13.31
Admin vs. IT	-9.54	3.54	-.12	-2.70	**.007**	-16.49 –-2.60
Admin vs. Office assistants	-3.99	2.87	-.06	-1.39	.165	-9.62–1.65
Admin vs. Scientists	-4.29	2.98	-.06	-1.44	.151	-10.15–1.56
Admin vs. Others	-4.26	2.58	-.08	-1.65	.099	-9.32–0.80
Transformational	6.11	1.44	.30	4.25	**< .001**	3.28–8.93
Transactional	1.04	1.42	.04	0.73	.464	-1.75–3.84
Laissez-Faire	-0.05	0.95	-.003	-0.05	.958	-1.91–1.81
Destructive	-1.86	1.06	-.09	-1.76	.080	-3.95–0.22

^1^ Values in bold mark significant p-values at an alpha level 0.05.

## Discussion

The study presented herein was conducted to answer two research questions:

- What is the state of well-being for leaders and followers at a tertiary hospital in Germany? How does it differ between the three largest occupational groups (i.e. physicians, nursing staff and administrative staff)?
- How are perceived leadership styles and subjective well-being at this tertiary hospital associated with each other?

In the following sections, results of the study are summarised (4.1) under the heading of the respective sub-question and compared with the findings in the literature (4.2). After that, the perspectives for future studies as well as implications for workplace health management, e.g. workplace health promotion (WHP), are discussed (4.3) followed by methodological limitations.

### Summary of the study results

Concerning well-being, we found a mean WHO-5 score for leaders significantly higher than the score of followers. Followers were more likely to have a WHO-5 score ≤ 50 than leaders. In the group of the nursing staff, followers were more likely to have a WHO-5 score ≤ 50 than leaders. Referring to the association of followers’ perception of their direct supervisor’s leadership style and their well-being, we found that transformational leadership and transactional leadership are positively correlated with WHO-5, while laissez-faire and destructive leadership are negatively correlated with WHO-5. Several differences were found between the professional groups concerning these correlations. Transactional leadership held the highest correlation for therapeutic staff, whereas for destructive leadership this correlation was observed for the IT staff. For some occupational groups, correlations remained insignificant, such as transformational leadership for physicians and IT staff; transactional leadership for physicians, IT staff and office assistants; laissez faire leadership for physicians, therapeutic staff and office assistants; and destructive leadership for physicians. Most surprisingly, no correlation was significant for physicians, on the contrary, some of them were nearly zero. Concerning the regression analysis, transformational leadership determined the well-being in the regression analysis significantly and contributed to the variance explanation of well-being.

### Integration and comparison of the results with existing evidence on leadership style and well-being

Findings of the present study show a relevant burden in the interviewed groups in the hospital workplace. This result is in line with current research (e.g. [54–56]). Followers reported lower well-being scores than leaders. More specifically, the mean scores for leaders (*M* = 56.8) and followers (*M* = 52.1) are lower than the scores of the German general population in the 2012 European Quality of Life Survey (see Supplementary Table 2 [18], p. 60). In comparison with a Danish study from the health sector (elderly care sector, *M* = 66.47 [57] (p. 101)), the values for well-being were lower in the present study. Compared to the industry sector, the employees in the health sector studied had lower well-being scores. For example, Feicht et al. [58] investigated the effects of web-based happiness training on psychological and physiological parameters in a German occupational health setting with employees of a local insurance company. The authors examined, among other things, happiness, satisfaction and quality of life in t0, t1 and t2. The authors found WHO scores around M ~63 in t0. Please note that we multiplied the WHO scores of Feicht et al. [58] by a factor of 5 to compare them with our results.

In our study, physicians without leadership responsibilities tended to have lower levels of well-being than other groups in our sample. These results are consistent with findings in the international literature, which provide impressive and consistent evidence of high rates of burnout among physicians (e.g. [56, 59]). Shanafelt et al. [56] found at least one burnout symptom of the dimensions of emotional exhaustion, depersonalization and low personal performance in over 50% of US physicians. *The Lancet* reports a "global crisis" in the title of its editorial regarding the high rates of burnout and its associated consequences [60]. The British Medical Association’s 2019 self-report survey found that 80% of participating doctors were at risk of burnout, with younger doctors—presumably lacking leadership responsibilities—at higher risk [60].

The higher scores for leaders´ well-being in our study are consistent with the findings of Klein et al. [54]. The authors investigated the relationship between experienced psychosocial work stress and perceived health care among surgeons in different hospitals in Germany. This result could be related to the higher perceived control and greater decision-making freedom of leaders compared to non-leaders. For example, O’Connor et al. [55] found that higher levels of control were associated with lower depression scores. Questionnaires assessing mental health, job satisfaction, psychological job demands, and job control were randomly distributed to general practitioners (GPs) and staff in other departments in the north of England. GPs were significantly more depressed and less satisfied with their work than staff in other departments. GPs who experienced high demands and low job control or low demands and low job control were particularly depressed.

In our study, amongst the professional group of nurses, overall WHO-5 scores tended to be lower (see above) than in in other occupational groups of this study. A review paper [61] examined the prevalence of burnout in mental health nurses to identify predictors of burnout. In most cases, the literature reports moderate levels of emotional exhaustion, depersonalization, and a feeling of being personally overwhelmed. The studies indicate that variables such as work overload, work-related stress, increased seniority, male gender, being single and workplace aggression, among other factors, contribute to the development of burnout. From a human resource development and safety perspective, it is important that leaders address the aspects of emotional exhaustion and low personal performance related to burnout reported by mental health nurses in the workplace.

Overall, the results of this study underpin the alarming situation of mental health in the hospital workplace, especially with regard to employees without leadership responsibility. The higher scores for leader well-being in our study are consistent with previous research that argues that leaders benefit from higher perceived control and the greater decision-making autonomy of leaders compared to that of followers.

A meta-analysis by Montano et al. [12] provides strong evidence that leadership is related to followers’ well-being. Leadership behaviour is an important determinant of employees’ occupational health in the workplace. Leadership style and the relationship between leader and followers act as both preventive and risk factors for employee mental health. Overall, destructive leadership is associated with poor mental health. Transformational leadership, high-quality relational and task-oriented leadership behaviours, and high-quality leader–follower interactions are positively associated with mental health. Overall, our findings are consistent with international evidence that leadership behaviour is associated with follower well-being (e.g. [12, 28, 62]). Arnold [28] conducted a systematic review of 40 studies between 1980 and 2015 to examine whether transformational leadership predicts employee well-being. Our results match the authors’ main finding: transformational leadership is highly correlated with follower well-being. Another systematic review also found a direct association between well-being and leadership styles on employees´ stress and affective well-being. Transformational leadership was positively associated with follower well-being [30]. Our result of impaired correlation of well-being and laissez-faire leadership is in line with Avolio [33] and an impaired correlation with destructive leadership is in line with Schyns and Schilling [25]. In their meta-analysis of 57 studies, the authors examined different conceptualizations of destructive leadership and the correlation between destructive leadership and various outcome variables. They found the strongest correlation between destructive leadership and attitudes towards the leader, with the second strongest correlation being between destructive leadership and counterproductive working behaviour.

Our finding that the well-being of physicians is not correlated with leadership behaviour represents the most significant–and at the same time intriguing–contradiction to the previous data. A possible explanation could be that other factors may play a more important role, since physicians often work without direct leadership contact. Here, there is already evidence from the literature that the relationship between transformational leadership and well-being is mediated by high levels of other working conditions or a balance of power in relationships in different occupational groups (e.g. [28]). Also, the variable "meaningfulness of work" [63] could mediate well-being or, for example, a high percentage of administrative tasks [64]. These possible influencing factors and assumptions could be investigated in further studies in the relevant population of physicians.

This importance of other working conditions for employee well-being may also explain the rather low variance explanation of leadership behaviour with regard to employee well-being. Other possible factors known from the literature could be these: time pressure, long working hours, general insecurity, frustration about how the job has to be done and social stressors with patients depending on the specialty [65]. Further factors could be hospital ownership, job requirements and job satisfaction [66]. In addition, transformational leadership made the highest contribution to the well-being of employees. This finding is in line with the concept of transformational leadership and previous research (e.g. [28, 30, 63]).

Altogether, our results are consistent with international findings that leadership behaviour is associated with the well-being of those being led (e.g. [12, 28, 62]). The most significant and challenging contradiction to the previous data is our finding that physician well-being does not correlate with leadership behaviour. On the other hand, the result that transformational leadership contributed the most to employee well-being is in line with the concept of transformational leadership and previous research (e.g. [28, 30, 63]).

### Implications and further directions

The present study contributes to the recording of mental health impairment of leaders, which is still an under-researched area (see [67]). Not only leadership but also other psychosocial working conditions play important roles in the well-being of healthcare workers (on this: [68]). It is therefore important to anchor the social dimension theoretically in stress models as well. One model that has already incorporated the social dimension is Karasek´s job demand–control (JDC) model [69, 70]. This model was extended by Johnson et al. [71] by adding the dimension of social support (S) from colleagues and leaders. Based on this adapted model, a lack of social support in the workplace increases the risk of psychological distress associated with low job control and high job demands. To reduce job stress, either workers’ subjective control would have to be improved or social support at work would have to be increased, according to the JDC-S model.

Upcoming studies might address the following issues and contexts in more detail. First, to assess work-related mental health, it may be important not to use an overarching concept such as well-being. Perhaps a more work-related concept such as irritation [72, 73], would be more useful as a "sensitive indicator of mental workload". Irritation is described as a state located between mental fatigue and mental illness. The development of the questionnaire is based on the transactional stress model of Lazarus (1966). The questionnaire consists of two scales: (1) cognitive irritation and (2) emotional irritation [72, 73]. The concept of irritation has already been used in the German health sector [74]. Second, variability in assessed leadership outcomes could be added. For example, leader–member exchange (LMX) theory as one other supportive leadership approach was recorded day by day in the study of Ellis et al. [75]. In their study, LMX theory is seen as a dynamic, interpersonal resource in the form of social support that is subject to daily variability, rather than a stable phenomenon. Results suggest that perceptions of LMX quality change on a daily basis. By recording the relationship between TFL and employee stress on a daily basis, variations in leadership behaviour can be accounted for. Again, it is found that leadership behaviour can vary from day to day [76]. Third, a future research project might involve measuring the relationship between leadership and employee well-being in times of very high psychosocial stress in the healthcare workplace–such as the COVID-19 pandemic–and comparing differences in that relationship and also in the assessment of well-being and leadership in the workplace (e.g. [77–79]).

Leaders in the health care sector, specifically in hospitals, should practice transformational leadership to foster employees’ well-being. As transformational leadership was only in the lower normative range interventions are needed to foster transformational leadership behaviour [45]. But health-oriented leadership interventions in the healthcare sector are scarce [80] and show mixed results. Single interventions with transformational leadership interventions in adjacent working areas show promising results [81], although recent meta-analysis and review could only find mixed results and no clear effect for employees’ well-being [80, 82]. This could be due to the fact that until now interventions have never targeted a whole organization, such as an entire hospital, but have rather been limited to single units [83] or single professional groups (e.g. [84]). Because of the complex work environment of hospital complexes, intervention approaches are needed to target employees’ well-being.

Such interventions should also contain specific parts which aim at an improvement of transformational leadership as well as elements of behavioural and structural prevention like the SEEGEN project [10]. There have been interdisciplinary and transdisciplinary research collaborations addressing the topic of mental health for employees in small and medium-sized enterprises (SMEs) since 2017 and 2020 in Germany. For instance, the research project IMPROVE*job* (see [85]) aims to reduce mental stress in the working life and increase job satisfaction and individual coping resources of team members in family practices. The project KMU-GO! (see [86]) evaluates a stress management programme for leaders, which includes units for individual stress reduction as well as a training part for stress-preventive leadership. Examples of such projects (e.g. SEEGEN, IMPROVE*job* and KMU-GO!) can help identify insights into the effectiveness of stress-preventive leadership in different organisational contexts as one essential aspect of workplace health management.

### Limitations

Results were taken from a prospective cohort study, which was conducted as a cross-sectional and partly explorative study (refer to research question 1). Therefore, the causal relationship between leadership and well-being can be explorative as suggested by the statistical methods used but cannot be sufficiently interpreted. Therefore, an associative relationship has to be interpreted. To further investigate actual causal relationships, future longitudinal research with similar cohorts is necessary. Moreover, we do not know if results are transferable to other sectors. The survey was conducted at a representative tertiary hospital in Germany, nevertheless the response rate with 11.26% had to mention as a possible limitation. If the response rate were calculated not on the number of email addresses but on the number of people who actually read that email, it would presumably be higher since not all addressees will have opened the email due to sick leave, parental leave or other reasons for absence from work. The number of opened emails is, however, unknown to us due to technical reasons. The final sample consisted of 1137 employees, including 27.7% who identified themselves as leaders and 72.3% who described themselves as followers. Future studies potentially enhance representativeness if conducted across several hospitals in a multicentre survey approach. Leaders and followers were interviewed using self-assessment instruments at one time point in a fast-changing working area, a method which is always open to biases. Needs and demands determined by quantitative methods could be supplemented by qualitative methods, such as single interviews or focus groups. Also, both comprehensive and individual activities and experiences could be depicted in more detail and potential programmes could be designed more effectively [87]. Finally, no information on the underlying mechanism of the relationship between leadership and well-being can be derived from the results of this study.

## Conclusion

Healthcare professionals are a particularly vulnerable group for work-related mental strain due to their high occupational psychological stress. With their work, the authors contributed to the current research process on health in different occupational groups and the influence of leadership style in the hospital workplace. The results showed that the relevant professional groups are under strain. Transformational and transactional leadership styles have a positive relationship with the general well-being of certain professional groups. Indispensable needs are supporting hospital staff in taking care of their own health, helping to form constructive interpersonal and interprofessional working relationships, and using their practical knowledge and experience to improve organisational working conditions.

The occupational group of hospital physicians should be examined more closely with more comprehensive methods regarding the relationship between health and leadership style with randomised controlled trials (RCT). The results also underline the need for more research into quantitative mapping and measurement of mental health and the addition of qualitative methods. Furthermore, the results reinforce the need for future multimodal workplace health promotion (WHP) and leadership interventions with behavioural and organisational intervention modules specifically tailored to hospital staff at different hierarchical and functional levels. However, preventive measures within the hospital have so far only targeted either individual occupational groups, sub-areas or organisational sub-units within a hospital and not the entire hospital. Finally, the results complement the general awareness of the need for effective workplace health management in hospitals in order to do justice to the complex mechanisms of stress-preventive leadership and health in the hospital workplace.
